# Case of Acute Petechiæ Hæmorrhagicæ

**Published:** 1823-04

**Authors:** William Pretty

**Affiliations:** Surgeon.


Art. III.—*Case of Acute Petechiæ Hæmorrhagicæ.* By William Pretty, Esq. Surgeon. (Communicated through Dr. James Johnson.)

Clarissa Bye, aged nine years, became the subject of the above disease. At the age of three years she left England for the West Indies, and returned in July 1822. Her mother considered her to have been subject to cough and pectoral disorder for some months, yet not in so severe a degree as to have caused any alarm: in every other respect, she seemed to have been as healthy and as strong as children in general.

*February* 1, 1823.—She appeared a little unwell; and, on Sunday, (the following day,) was attacked with sickness, severe pain at the stomach and the region of the navel, and with some thirst and fever. The tongue was much furred; the pulse accelerated, but not full or hard; the respiration was quickened; and she had also some pain in the head. Every thing taken had been thrown up immediately, up to the time of my first seeing her. The bowels had been opened the day before; but, wishing to have them more freely relieved, I prescribed colocynth and calomel pills, with extract of poppy.

*Monday, the 3d.*—The pills not having operated otherwise than apparently in stopping the vomiting, which was considerably lessened, a cathartic mixture was given, which procured several fetid evacuations. In the middle of the day I saw her again, and found her with still the same pains in the stomach and head; the face was suffused with redness, and the tunica conjunctiva red and vascular; the insides of the hands were of a dark red colour, like the face, but no where else was it perceptible; the breathing was more frequent, accompanied with a troublesome cough, and the fever altogether increased. She was bled by my apprentice, to the amount of six ounces only, in consequence of some obstruction occurring to the free flow of blood. As the sickness had so much abated, some nitrate of potass, and antimonial powder, were prescribed. Some petechiæ, of the size of pins' heads, were now observed about the arms, breast, and abdomen, and a few upon the right leg. Upon the fore and middle part of the left leg, there was a large blue spot under the cuticle, of the size of a sixpence, without any very apparent separation of that skin from the cutis. Some sanguinous discharge appeared upon the inside of the labia pudendi.

*Tuesday the 4th.*—Has passed a very restless night, the fever having very considerably increased. The functions of the lungs were very much impeded, threatening suffocation; the brain was suffering under the same violent determination of blood; the pulse rapid, hard, and inflammatory; the cough not very violent, but a very frequent retching without any thing being brought up; the bowels were quite free; the petechiæ had increased in number, particularly upon the back part of the left hand, which was very thickly spotted; the purple spot on the left leg had increased one-third in circumference. Mr. Bagster saw her with me, and we agreed upon bleeding her again, as being the most likely means of preserving the head and lungs from serious injury. Ten or twelve ounces of blood were taken from the arm, which produced syncope and a great change in all her symptoms, promising much benefit. A few hours after, fever again appeared, and Dr. James Johnson, who saw the patient in my absence, approved of the measures pursued, and recommended the exhibition of the mineral acids. In the evening, symptoms of sinking supervened; she had all the appearance of a person dying, or in the last stage of pulmonic inflammation. At eight o'clock next morning (Wednesday,) she expired.

The blood first drawn exhibited no serum for the space of about eight hours; but on the next day, after twenty hours' standing, a small quantity appeared. One teacupful of the second bleeding showed a coat of coagulable lymph half an inch thick, like very soft jelly, and with the crassamentum very loose, and so tender as to be broken down very easily with a spoon into a soft pulpy mass. In this cup there was little or no serum. The other portions of blood, which were not taken with an uninterrupted stream, showed no buffy coat and very little serum: it seemed as though the blood had become so dissolved or altered as to be unable to separate in the usual way. Upon what this change in the vital fluid could depend, I cannot tell: it is clearly a principal morbid feature in this complaint, and probably the cause of some of the violent symptoms which were present in this case. The stomach was the organ first affected, and, as will appear by dissection, exhibited more striking marks of the disease than any other viscus in the body.

*Post-mortem Examination.*—In addition to the facts above mentioned, the eyelids were thickly set with petechiæ, and many of a larger size appeared upon the back of the trunk. In the brain nothing, materially differing from health, was observed: upon slicing it, many bloody points appeared; the veins of membranes were more distinct than common; and, upon the whole, it showed traces of inordinate determination to its vessels. In the thorax, we found adhesions between the pleura costalis and pulmonalis in the left cavity, the common result of inflammation of that membrane: they were, however, not of recent formation. The lungs themselves, though not so heavy as to sink in water, seemed unnaturally loaded with blood and mucus, and therefore felt and appeared more firm than in a healthy state: in other respects they were sound. The pericardium contained nearly half an ounce of liquor. The external surface of the heart of a pale hue, and about twenty small petechiæ on various parts of it, but more particularly at the junction of the auricles and ventricles. In the abdomen, at first all appeared natural, with the exception of the stomach: it was distended with air, and was thickly spotted with petechiæ, which were very plainly seen through its peritoneal and muscular coats; and, upon opening that viscus, they were, of course, much more distinctly seen, of about the size of split-peas generally; some, however, were smaller, and were situated in the substance of the villous or mucous coat. The stomach contained about three ounces of a greenish fluid. The intestines were cut open in several places, but no petechiæ were seen upon their internal surface. A number of the mesenteric glands were enlarged, and were of a dark-purple colour.—Dr. Gregory and Mr. Bagster were present at this examination.

*Mabledon Place; 14th February*, 1823.

